# Identification of Differentially Expressed Genes in BALB/c Mouse Liver upon Primary Infection with DENV1 and Sequential Heterologous Infection with DENV2

**DOI:** 10.3390/pathogens7040078

**Published:** 2018-10-02

**Authors:** Indeevari A. C. Wickremsinghe, Vinod R. M. T. Balasubramaniam, Y. Yik Mot, Amreeta Dhanoa, Sharifah S. Hassan

**Affiliations:** 1 Jeffrey Cheah School of Medicine and Health Sciences, Monash University Malaysia, Jalan Lagoon Selatan, Subang Jaya 47500, Selangor, Malaysia; iawic1@student.monash.edu (I.A.C.W.); yymot@hotmail.com (M.Y.Y.); amreeta.dhanoa@monash.edu (A.D.); 2 Tropical Medicine & Biology Multidisciplinary Platform, Monash University Malaysia, Jalan Lagoon Selatan, Subang Jaya 47500, Selangor, Malaysia

**Keywords:** DENV 1, DENV 2, annealing control primer, differentially expressed genes, apoptosis, IgG, IgM

## Abstract

Dengue virus (DENV) results in 100 million cases of infections and 22,000 deaths per year. Liver involvement, thrombocytopenia, haemorrhage and plasma leakage are characteristic manifestations of severe forms of DENV infection. However, the molecular pathways of DENV infection have not been comprehensively studied compared to the host immunological responses. We performed an in vivo study using the BALB/c mouse model with a modified mRNA differential display methodology (GeneFishing^TM^) using the annealing control primer (ACP) system to capture differentially expressed genes (DEGs) in mice liver upon primary infection with DENV1 and sequential heterologous infection with DENV2. Secondary heterologous infection with DENV2 was carried out at Immunoglobulin IgM and IgG peaks following the primary DENV1 infection with the hope of determining any potential effect antibodies IgM and IgG may have on sequential heterologous infection. 30 DEGs were identified and sequenced across all three treatment groups and they belong to a variety of important pathways such as apoptosis, innate immune response, inflammatory response, metabolic processes and oxidative stress. Analysis of differentially expressed genes in response to viral infection offers valuable knowledge about the dynamic and complex association between host cell and the virus. Furthermore, some DEGs identified support DENV induced liver damage.

## 1. Introduction

Dengue is a vector-borne virus, transmitted to humans via infected mosquitoes; primarily by *Aedes aegypti* and *Aedes albopictus*, in tropical and sub-tropical areas [1,2]. Eighty percent of infected individuals remain asymptomatic or experience uncomplicated dengue fever (DF); which is an acute febrile illness accompanied by retro orbital pain, headache and myalgia. Dengue hemorrhagic fever (DHF) and dengue shock syndrome (DSS) are the life-threatening forms of dengue virus (DENV) infection and are more commonly associated with a secondary dengue infection [3,4]. The clinical features of DHF include liver enlargement and dysfunction, thrombocytopenia, hemorrhage and plasma leakage [5]. Severe cases of DHF have been known to progress to DSS [6]. DSS is a form of hypovolemic shock due to plasma leakage into interstitial space which is clinically associated with hemoconcentration, and may be fatal if the patient fails to receive appropriate care [7]. Currently, for patients with DHF and DSS, aggressive intravenous fluid replacement therapy is the only form of treatment available [8].

A significant body of clinical and experimental evidence has implicated the involvement of the liver during DENV infection and the liver as a critical component of the disease pathology [9,10,11,12]. One of the most prominent signs indicating liver involvement during DENV infection is liver enlargement or hepatomegaly, and is observed in a significant proportion of dengue cases [9,13]. Another indicator is the up-regulations in hepatic enzymes aspartate aminotransferase (AST) and alanine aminotransferase (ALT), which are sensitive indicators of liver damage, during DENV infection and have been more commonly observed in patients with DHF/DSS than with DF [9,12,14,15]. 

To date, in vitro host gene expression profiles of different cell lines infected with one DENV serotype have been studied but, the significance of the altered gene expressions observed during infection however remains to be determined in vivo. Additionally, no study has investigated the in vivo broad gene expression changes in liver cells in response to sequential heterologous DENV infections, despite significant evidence demonstrating that the virus replicates in the liver and prominent association between the liver and DHF/ DSS [16]. In this study, we carried out differential gene expression studies of liver: (1) upon primary DENV infection and (2) sequential heterologous DENV infection in vivo using an improved mRNA differential display technique, GeneFishingTM using Annealing Control Primers to accurately identify differentially expressed genes (DEGs), since detection of authentic DEGs would allow for a better understanding of the complicated mechanisms and pathways involved during primary and sequential heterologous DENV infection.

## 2. Results

**Figure 1:**
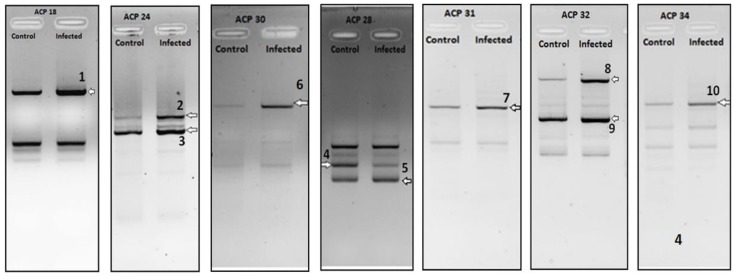
DEGs Sequenced from liver samples of BALB/c primary infected with DENV1. Arrow indicates differential cDNA bands and each number indicates the differentially expressed gene (DEG) number.

**Figure 2:**
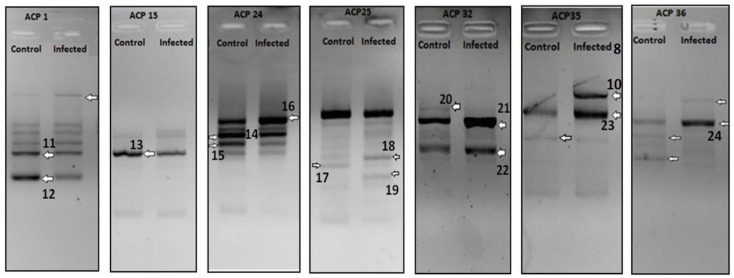
DEGs Sequenced from liver samples of BALB/c sequentially infected at IgM peak. Arrow indicates differential cDNA bands and each number indicates the DEG number.

**Figure 3:**
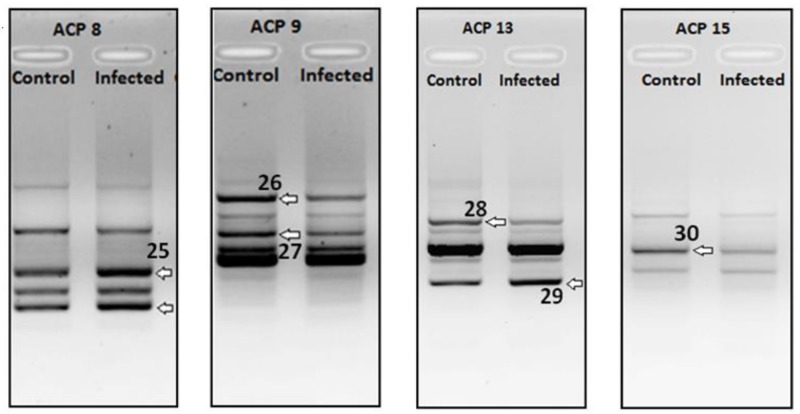
DEGs Sequenced from liver samples of BALB/c sequentially infected at IgG peak. Arrow indicates differential cDNA bands and each number indicates the DEG number.

From the generated data of 30 identified and sequences DEGs (Figure 1, Figure 2 and Figure 3), a comparative analysis was made based on the number of up-regulated and down-regulated genes observed for each of the treatment groups. 

The selected bands were isolated, cloned in TOPO2.1 vector (Invitrogen, Carlsbad) and sequenced to confirm their identity. As can be seen from Table 1, 10 genes were differentially regulated (3 up-regulated and 7 down-regulated) in for livers of primary DENV1 infected mice. 

A total of 14 genes were observed to be differentially regulated (7 up-regulated and 7 down-regulated) in livers of mice that underwent sequential heterologous infection at the IgM peak (Table 2). 

Finally, a total of 6 genes were differentially expressed (2 up-regulated and 4 down-regulated) in livers of mice that underwent sequential heterologous infection at the IgG peak (Table 3). 

**Figure 4:**
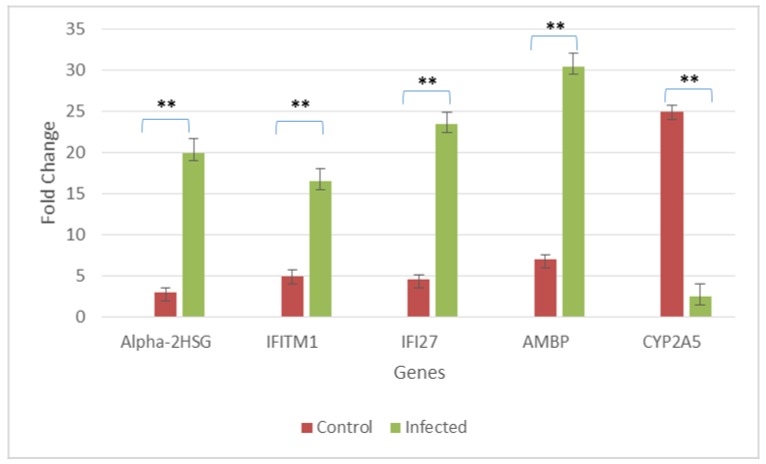
Confirmation of regulation selected genes from control and infected mice liver. Transcript levels analyzed using quantitative real-time polymerase chain reaction (qRT-PCR) reveal similar expression with the mRNA differential display. RNA from control mice liver and DENV1/DENV2, primary and sequential heterologous infected mice liver at IgG and IgM peaks were subjected to real time PCR and fold change was calculated accroding to 2^−ΔΔCT^ method and results from the real-time RT-PCR were analyzed by two-tailed Student’s *t* test. A *p* value ≤ 0.005 was considered to be statistically significant (shown as ** in the figure).

From this, it can be seen that there were clear differences in the response elicited by liver cells in primary and sequential infection. Furthermore, based on the analyses of liver samples collected at the two stages studied during heterologous infection which demonstrated significant differential up-regulation and down-regulation of several gene families, it can be inferred that liver response to sequential infection at the IgM peak and IgG peak were distinctly different. Additionally, the proteins coded by the identified DEGs also play important roles during infection such as apoptosis, inflammatory response and viral replication. We further confirmed our results using quantitative real time PCR. From our mRNA differential display studies, we identified five genes which were involved DENV related liver damage; Alpha-2HS-glycoprotein, IFITM1, IFI27, AMBP and CYP2A5 to confirm their expression pattern correlates with our mRNA differential display PCR. Figure 4 shows the results obtain with all of these genes to follow similar pattern with differential display PCR. 

## 3. Discussion

Sequential heterologous infection results in more severe DENV infection and hence greater liver damage. Based on our results, we have identified a gene that was differentially expressed between primary and sequential infection that is postulated to result in this scenario. Humanin was seen to be up-regulated during primary infection but down-regulated during sequential heterologous infection at the IgM peak. Since Humanin is responsible for negative regulation of apoptosis, down-regulation in sequential infection potentially reflects a viral adaptation to escalate apoptosis and thereby causing greater liver damage. Furthermore, the sequential heterologous DENV infection at the IgM and IgG peaks also provided additional insights into the roles of infection associated mediators and potential leads for future studies into the severe forms of dengue infection. A few genes identified during the IgG peak such as IFI27 (up-regulation), S-formylglutathione hydrolase (up-regulation) and Ribosomal Protein L18a (down-regulated) seem to be regulated in a manner to potentially confer a degree of protection from the sequential infection in BALB/c mice. On the contrary, a majority of genes sequenced at the IgM peak highlight progression towards greater liver damage and infection; such as Humanin (down-regulation), IFITM1 (down-regulation), CYP2A5 (down-regulation), and Alpha-2HS-glycoprotein (up-regulation). 

Interestingly, some of the genes previously associated with DENV pathogenesis were also found in this study (e.g., Alpha-2HS-glycoprotein, IFITM1, IFI27, and AMBP), thus supporting the potential link of these identified novel genes to DENV infection. It has also been previously established that DENV in vivo results in the differential regulation of metabolic genes [17]. In this study, several novel genes involved in cellular metabolic processes (nucleic acid metabolism—nuclear receptor subfamily group 1 member 2; protein metabolism—prefoldin-5, TOM6, ribosomal protein L12 and L18a; lipid biosynthesis—estradiol 17 beta dehydrogenase 11) were identified to be involved during DENV infection of mice livers. Elucidation of the roles of such genes during DENV infection might provide new insights into DENV pathogenesis and disease progression. As such it can be concluded that identification and validation of a broader range of DEGs might pave paths to deduce the effects of DENV on the liver and hopefully elucidate differences in the pathophysiology of severe versus non-severe DENV infection in humans. Conclusively, through this system, we have identified and sequenced 30 DEGs which were determined to belong to a variety of important pathways such as apoptosis, innate immune response, inflammatory response, metabolic processes and oxidative stress. It is postulated that this method of DEG determination could be applied in a wide range of pathologies including infectious and genetic diseases to elucidate the mechanisms and progression of the conditions.

## 4. Materials and Methods

### 4.1. Viruses

Dengue virus (DENV) serotypes 1 and 2 (DENV1: Genbank - FR666924.1; DENV2 - Genbank: AJ556804.1) used in this study were isolated from human serum samples which were a generous gift from Professor Sazaly Abu Bakar of Tropical Infectious Diseases Research and Education Centre (TIDREC), University of Malaya, Malaysia. DENV1 and DENV2 stocks were propagated in Vero cells in Minimal Essential Media (containing 2% FBS; GIBCO) to obtain high viral titers. All virus stocks obtained were stored at −80 °C until further use. Using the Beckman Coulter OptimaTM LX-100 Ultracentrifuge, DENV 1 and DENV 2 stocks were concentrated at 30,000 rpm for 3 h at 4 °C. Virus pellets obtained were resuspended in 1× MEM and the resultant concentrated virus solutions were kept at 4 °C for use the day after [18]. Concentrated DENV1 and DENV2 solutions were titrated via TCID_50_ assay as described previously [19] and were calculated to be 1.26 × 10^7^/mL and 7.94 × 10^6^/mL respectively.

### 4.2. Animal Work

All experimental protocols were performed in accordance with the guidelines for animal research of the Monash University and this study was approved by the said Institutional Animal Care and Use Committee and carried out according to the Animal Ethics Australia: MARP/2012/114 Four-week-old female BALB/c mice were used in this study. The mice were inoculated with DENV via both intravenous (I.V.) and subcutaneous (S.C.) routes. Mice were infected with one serotype of DENV (DENV1) to represent a primary infection, and also with two heterologous serotypes (DENV1 followed by DENV2) to represent a sequential heterologous infection, in order to determine differences in hepatic gene expression between primary and sequential heterologous DENV infected mice. The mice were housed individually in mosquito proof cages. Livers obtained during the course of the experiment were from the following three treatment groups: (1) Primary (DENV1) infected mice, (2) Sequential heterologous (DENV2) infected mice at IgM peak, (3) Sequential heterologous (DENV2) infected mice at IgG peak, and from their respective (4) mock-infected (control) mice counterparts of (1), (2) and (3). Thirty milligrams of mice liver was homogenized, transferred to a sterile 1.5 mL microfuge tube (Eppendorf) and RNA was extracted using the SV Total RNA Isolation System (Promega).

### 4.3. mRNA Differential Display PCR

First strand cDNA was synthesized based on a previously designed methodology by Hwang et al., 2003 using the M-MLV reverse transcriptase (Promega). Reverse transcription was performed at 42 °C for 90 min, followed by 94 °C for 2 min. First-strand cDNA was stored at −20 °C until use. Second strand cDNA synthesis and subsequent PCR amplification was carried out in a final reaction volume of 49.5 μL containing 3–5 μL (amounting to 50 ng) of diluted first-strand cDNA, 5 μL of 10× Dream Taq reaction buffer (Fermentas) 1 μL of 10 mM dNTP, 1 μL of 10 μM dT-ACP2, and 2 μL of a 5μM arbitrary primer. The reaction mixture was held at 94 °C, while 0.5μL of 5 U/μL Dream Taq Polymerase (Fermentas) was added to the reaction mixture. The PCR protocol for second strand synthesis was one cycle at 94 °C for 1 min, followed by 50 °C for 3 min, and 72 °C for 1 min. Following the completion of second-strand DNA synthesis, 40 cycles were performed. Each cycle involved denaturation at 94 °C for 40 s, annealing at 65 °C for 40 s, extension at 72 °C for 40 s, and a final extension at 72 °C for 5 min to complete the reaction. The amplified PCR products were separated in 2% agarose gel stained with ethidium bromide. The exact procedure was repeated with all cDNA samples from the three treatment groups and their respective control counterparts using the provided ACPs to amplify the DEGs [20,21,22].

### 4.4. Quantitative Real-Time PCR

Quantitative real-time PCR was done according to our previous methods established in our lab. Briefly, PCR reactions were set up in 96-well optical plates using 50 ng of extracted control mice liver and infected RNA from DENV1/DENV2, primary and sequential heterologous infected mice liver at IgG and IgM peaks, 10 μL TaqMan Universal PCR Master Mix (Applied Biosystems, Foster City, CA, USA), and 1 μL of primers/probe set containing 900 nM of forward and reverse primers and 300 nM probe to a final volume of 20 μL per reaction in triplicates [19,20]. RT-PCR program consisted of incubation at 48 °C for 30 min, and 40 cycles at 95 °C for 10 min, 95 °C for 15 s, and 60 °C for 1 min with the Step One Plus Real-Time PCR System^®^ (Applied Biosystems). A non-template control and an endogenous control (eukaryotic 18 s rRNA) were used for the relative quantification. All quantitations (threshold cycle [CT] values) were normalized to that of 18 s rRNA to generate ΔCT, and the difference between the ΔCT value of the sample and that of the reference (uninfected sample) was calculated as ΔΔCT. The relative level of gene expression was expressed as 2^−ΔΔCT^ [20,21,22,23]. Full sequence of primers used will be provided upon request to the authors.

## Figures and Tables

**Table 1:** DEGs identified from livers of Primary (DENV1) Infected BALB/c mice.

DEG No.	Regulation (Infected)	Identified mRNA	Max Identity	Accession
1	Down	Mus musculus kelch-like 25 (Drosophila), mRNA (cDNA clone IMAGE:4159269)	99%	BC022600.1
2	Down	Mus musculus histidine ammonia lyase, mRNA (cDNA clone MGC:67748 IMAGE:4211545), complete cds	99%	BC057637.1
3	Down	Mus musculus cDNA sequence BC024137, mRNA (cDNA clone IMAGE:5136153), partial cds	99%	BC024137.1
4	Down	Mus musculus SEC62 homolog (S. cerevisiae) (Sec62), mRNA	99%	NM_027016.2
5	Down	Mus musculus prefoldin 5 (Pfdn5), mRNA	99%	NM_027044.3
6	Up	Mus musculus translocase of outer mitochondrial membrane 6 homolog (yeast), mRNA (cDNA clone MGC:46796 IMAGE:5007624), complete cds	98%	BC037589.1
7	Down	Mus musculus C-reactive protein, pentraxin-related, mRNA (cDNA clone MGC:18634 IMAGE:4195658), complete cds	100%	BC011124.1
8	Up	Mus musculus S-adenosylhomocysteine hydrolase (Ahcy), mRNA	99%	NM_016661.3
9	Up	Mus musculus alpha 1 microglobulin/bikunin, mRNA (cDNA clone MGC:14070 IMAGE:4193922), complete cds	99%	BC021660.1
10	Down	Mus musculus mitochondrial DNA from Lewis lung carcinoma, complete sequence	88%	AP013054.1

**Table 2:** DEGs identified from livers of BALB/c mice that underwent sequentially heterologous (DENV2) infection at IgM peak.

DEG No.	Regulation (Infected)	Identified mRNA	Max Identity	Accession
11	Down	Mus musculus fibronectin 1, mRNA (cDNA clone IMAGE:4985138), partial cds	99%	BC036167.1
12	Down	Mus musculus mitochondrial DNA from Lewis lung carcinoma, complete sequence	96%	AP013054.1
13	Down	Mus musculus mRNA for serum albumin	99%	AJ011413.1
14	Down	Mus musculus interferon induced transmembrane protein 1 (Ifitm1), transcript variant 2, mRNA	94%	NM_001112715.1
15	Down	Mus musculus ribosomal protein L12 (Rpl12), mRNA	99%	NM_009076.3
16	Up	Mus musculus MACRO domain containing 1, mRNA	78%	BC008653.1
		(cDNA clone MGC:11843 IMAGE:3597023), complete cds		
17	Down	Mus musculus capping protein (actin filament) muscle Z-line, beta	100%	NM_001271405.1
		(Capzb), transcript variant 3, mRNA		
18	Up	Mus musculus alpha-2-HS-glycoprotein (Ahsg), mRNA	83%	NM_013465.1
19	Up	Mus musculus hydroxysteroid (17-beta) dehydrogenase 11	99%	NM_053262.3
		(Hsd17b11), mRNA		
20	Down	Mus musculus cytochrome P450, family 2, subfamily a, polypeptide 5	99%	NM_007812.4
		(Cyp2a5), mRNA		
21	Up	Mus musculus alpha 1 microglobulin/bikunin, mRNA	99%	BC021660.1
		(cDNA clone MGC:14070 IMAGE:4193922), complete cds		
22	Up	Mus musculus cytochrome P450, family 2, subfamily d, polypeptide 26, mRNA	100%	BC023241.1
		(cDNA clone MGC:28632 IMAGE:4222538), complete cds		
23	Up	Mus musculus FCF1 small subunit (SSU) processome component homolog	97%	NM_028632.2
		(S. cerevisiae) (Fcf1), mRNA		
24	Up	Mus musculus pyrophosphatase (inorganic) 1, mRNA	99%	BC010468.1
		(cDNA clone MGC:6716 IMAGE:3585780), complete cds		

**Table 3:** DEGs identified from livers of BALB/c mice that underwent sequentially heterologous (DENV2) infection at IgG peak.

DEG No.	Regulation (Infected)	Identified mRNA	Max Identity	Accession
25	Up	Mus musculus esterase D/formylglutathione hydrolase, mRNA (cDNA clone MGC:57923 IMAGE:5694094), complete cds	99%	BC046766.1
26	Down	Mus musculus pregnane X receptor mRNA, complete cds	97%	AF031814.1
27	Down	Mus musculus hairy and enhancer of split 6 (Drosophila) (Hes6), mRNA	99%	NM_019479.3
28	Down	Mus musculus mitochondrial pyruvate carrier 1, pseudogene (Mpc1-ps) on chromosome 12	99%	NG_032669.1
29	Up	Mus musculus interferon, alpha-inducible protein 27 like 1 (Ifi271), transcript variant 5, mRNA	99%	NM_194069.2
30	Down	Mus musculus ribosomal protein L18A (Rpl18a), mRNA	81%	NM_029751.4
